# Interconnected study of molecular pathways: miR-137 as a central element at the intersection of lipid metabolism and prostate carcinogenesis

**DOI:** 10.31744/einstein_journal/2025AO1601

**Published:** 2025-08-08

**Authors:** Karina Serafim da Silva, Vanessa Ribeiro Guimarães, Feres Camargo Maluf, Gabriel Arantes dos Santos, Juliana Alves de Camargo, Iran Amorim da Silva, Katia Ramos Moreira Leite, Sabrina Thalita dos Reis, Nayara Izabel Viana, Miguel Srougi, Ruan Pimenta

**Affiliations:** 1 Hospital das Clínicas Faculdade de Medicina Universidade de São Paulo São Paulo SP Brazil Laboratório de Investigação Médica 55 (LIM55), Hospital das Clínicas, Faculdade de Medicina, Universidade de São Paulo, São Paulo, SP, Brazil.; 2 Department of Urology University of California San Francisco San Francisco CA United States Department of Urology, University of California San Francisco, San Francisco, CA, United States.; 3 Hospital Sírio-Libanês São Paulo SP Brazil Hospital Sírio-Libanês, São Paulo, SP, Brazil.; 4 Universidade do Estado de Minas Gerais Passos MG Brazil Universidade do Estado de Minas Gerais, Passos, MG, Brazil.; 5 Instituto D’Or de Pesquisa e Educação São Paulo SP Brazil Instituto D’Or de Pesquisa e Educação, São Paulo, SP, Brazil.

**Keywords:** Prostatic neoplasms, Lymph nodes, Lipid metabolism, Biomarkers, Disease progression, Prognosis, Disease-free survival, Health strategies

## Abstract

**Objective:**

To evaluate the roles of miR-137 and its target genes in lipid metabolism and prostate tumorigenesis.

**Methods:**

We used a series of bioinformatic approaches to establish the relationship between miR-137 and its target genes. We mapped the metabolic pathways of interest in the Reactome database and identified the central target genes of miR-137 in this pathway using four platforms: Reactome, miRDB, miRmap, and TargetScan. To assess the expression and association with clinical parameters, we obtained information from the UALCAN, OncoDB, and GEPIA2 databases using a dataset of patients with prostate cancer from The Cancer Genome Atlas. For functional enrichment analysis and construction of the protein-protein interaction network, we used the Kyoto Encyclopedia of Genes and Genomes, Gene Ontology, and STRING.

**Results:**

Our *in silico* study of The Cancer Genome Atlas database revealed that miR-137 is underexpressed in tumor tissues, and its reduction is associated with poor prognosis. An intriguing set of eight genes within the PPARα pathway: *PPARGC1A*, *PPARGC1B*, *NCOA1*, *NCOA2*, *NCOA3*, *MED1*, *MED27*, and *ESRRA* displayed synergy, positive correlations, and synchronized expression patterns in adipose, hepatic, and prostatic tissues, all linked to the enigmatic processes of metabolic regulation. Among the highlighted genes, *ESRRA* was overexpressed in the malignant environment, whereas its counterparts remained underexpressed. The plot was thickened with associations between the expression of *NCOA1*, *NCOA3*, and *MED27*, lymph node involvement, and the overexpression of several genes linked to advanced prostate cancer stages. An intriguing pattern emerged, with patients exhibiting reduced disease-free survival overexpressing *NCOA2*, *NCOA3*, *MED27*, and *ESRRA*.

**Conclusion:**

This study elucidates the possibility that miR-137 subtly modulates metabolic genes in prostate cancer, suggesting its latent therapeutic potential as a biomarker for disease progression.

## Highlights

■ The reduction of miR-137 in tumor tissues is associated with a worse prognosis.

■ miR-137 has eight oncogenically relevant target genes acting in the PPARα lipid pathway.

■ *NCOA1, NCOA3, MED27*, and *ESRRA* are associated with advanced prostate cancer.

■ miR-137 exhibits significant clinical potential by repressing the activation of pathways that influence prostate tumorigenesis in hyperstimulated metabolic environments.

## In Brief

Prostate cancer progression is sustained by the simultaneous activation of pathways involving lipid uptake and de novo synthesis. In this context, miR-137 inhibits adipogenic differentiation and may reduce lipid uptake by tumor cells by modulating the PPAR/ p160/ESRRA axis, considerably attenuating metabolic effects and suppressing prostate tumorigenesis.

## Graphical abstract

**Figure:**
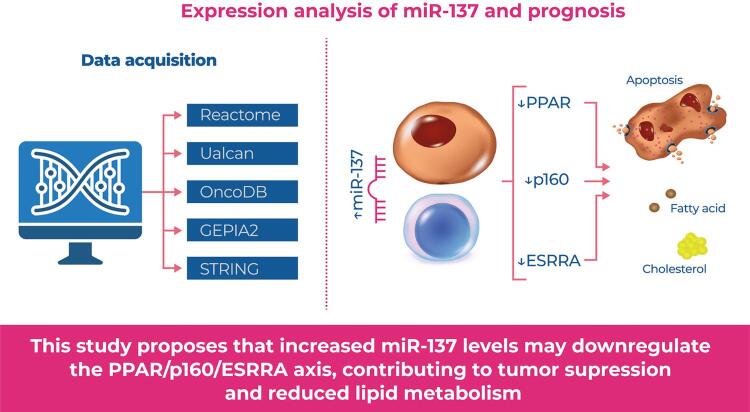

## INTRODUCTION

Prostate cancer (PCa) is a neoplasm with high incidence and prevalence among men.^(1)^ There are several challenges in effectively managing diseases. With the advent of molecular characterization, the search for molecules with clinical potential for diagnosis, prognosis, and therapy has become urgent, given that the prostate cancer microenvironment exhibits notable heterogeneity, manifests in various ways, and is influenced by epidemiological, genetic, and molecular factors. Although many cases of localized disease are treatable and curable, the scenario changes when indolent and lethal forms establish themselves without the opportunity for clinical stratification. Moreover, the lack of validated biomarkers reflects the challenge of treating prostate tumors, which are characterized by multifocal and heterogeneous cellular features that affect treatment responses.^(1)^

Androgen Deprivation Therapy (ADT) is the cornerstone of prostate cancer treatment, and its benefits reflect the role of androgens. Despite its effectiveness, most patients face the burden of ADT and develop Castration-Resistant Prostate Cancer (CRPC), which occurs in various ways, including metabolic reprogramming accompanied by the persistence of the androgen axis.^(2)^

Metabolic reprogramming is a hallmark of PCa tumorigenesis. In the presence of androgens, tumor cells undergo intratumoral synthesis and systemic lipid uptake to stimulate their growth.^(3)^ When coupled with the androgen receptor (AR), this hormone triggers an anabolic program by activating lipogenic and cholesterogenic genes. In addition, they are essential stimulators of the lipolysis pathway, the fatty acids generated by which are used by tumor cells as energy substrates.^(4,5)^It is widely recognized that androgen-mediated effects occur in the presence of cholesterol. Thus, positively regulating pathways involved in lipogenesis and cholesterogenesis through androgen signaling drives several processes, including proliferation, invasion, migration, and therapeutic resistance.^(6)^

Although molecular events are far from becoming a reality in translational aspects, the implementation of epigenetic regulators, such as microRNAs, as diagnostic, prognostic, and therapeutic tools is gaining momentum, especially in the context of prostate cancer.^(7-12)^ Their ability to target thousands of targets has emerged to overcome highly heterogeneous tumors and significantly regress disease progression. With prostate cancer, one of the primary therapeutic modalities is the inhibition of the androgen and cholesterol pathways.^(13,14)^ Coincidentally, miR-137 was predicted to target both scenarios. However, they are epigenetically silenced in patient tumors due to methylation events in the DNA regions they encode. In the androgen-responsive cell line LNCaP, it is hemimethylated, whereas in non-responsive cells, such as PC-3, it is hypermethylated, which comprehensively affects its modulatory function.^(12)^ Recently, we demonstrated the influence of a hypercholesterolemic microenvironment on the regulatory activity of miR-137 in a preclinical model of advanced metastatic PCa.^(15)^ miRNAs significantly reduced cell growth in the group supplemented with a hypercholesterolemic diet compared to that in the group fed the control diet. Although conflicting, in advanced disease, there is an increase in lipid pathways and cholesterol levels to sustain progression in the absence of androgens. Therefore, considering that the above *in vivo* model was established using a cell line representative of advanced disease, the increase in cholesterol levels significantly contributed to the restoration of miR-137 in the hypercholesterolemic diet group. Given that miR-137 usually responds to the presence of androgens or their precursor cholesterol, it is pertinent to suggest that although methylation impacts its activity, the presence of androgens or their precursors partially contributes to restoring homeostasis in the cellular environment. In addition, miR-137 inhibits adipogenic differentiation,^(16)^ reinforcing its diverse roles in lipid pathways through molecular interactions.

Understanding the metabolic and tumorigenic roles of miR-137 may provide substantial clinical benefits, as sensitivity to ADT can be lost owing to secondary cellular metabolic reprogramming. Thus, understanding these associations sheds light on potential therapeutic targets and prevention strategies aimed at modulating lipid metabolism and consequently treating advanced forms of prostate cancer.

In this study, we used bioinformatics tools to analyze the patterns of gene expression and miR-137 levels in prostate cancer samples. We propose that miR-137 is a potential key regulator of prostate cancer progression and proliferation owing to its modulation of lipid metabolism by targeting crucial genes associated with this pathway, particularly in patients with prostate cancer. Ultimately, this intricate regulation may be related to tumor progression and therapeutic response failure, adding a crucial dimension to our understanding of the molecular mechanisms underlying prostate cancer.

### OBJECTIVE

We aimed to evaluate the impact of miR-137 on the regulation of a set of genes associated with lipid pathways and prostate cancer pathogenesis and to understand, from a molecular perspective, the potential influence of tissues responsible for cholesterol synthesis and metabolism on prostate cancer progression.

## METHODS

### Expression analysis of miR-137 and prognosis

To evaluate the miR-137 expression profile in PCa and its association with clinicopathological factors such as the Gleason score and nodal metastasis status, we used expression values from the TCGA database, which is available at the University of Alabama at Birmingham Cancer Data Analysis Portal online platform.^(17)^ Expression values are based on miRNA-seq and correspond to pre-miRNA regions. The TCGA population comprised 490 primary tumor samples and 51 adjacent normal tissue samples. All images were generated using UALCAN software.

### Target prediction and expression profile based on tissue type

To predict the miR-137 target genes involved in lipid metabolism, we first used the Reactome 88.0 platform to determine the metabolic pathway.^(18)^ Reactome database has a hierarchical arrangement ranging from basic to more complex interactions. Thus, we filtered our searches based on lipid metabolism and defined the pathways associated with the regulation of lipid metabolism by PPARα. To predict miR-137 target genes involved in regulating lipid metabolism by PPARα, we exported the gene tables from the Reactome 88.0, miRDB 6.0, miRmap, and TargetScan 8.0 databases.^(18-21)^ We imported it into the Bioinformatics & Evolutionary Genomics platform^(22)^ to illustrate the Venn diagram and determine equally predicted genes in the four databases. Before prediction analysis, we used the Expression Atlas platform to evaluate gene expression patterns among the RNA-seq-based human tissue samples.^(23)^ To better understand the similarities or divergences between lipid metabolism and utilization, we selected three of the 32 available tissues: the liver, adipose, and prostate. Next, we determined the metabolic pathway of interest in Reactome,^(18)^ redirected our analysis to the Expression Atlas using the pathway ID R-HSA-400206 as a command, filtered the tissues of interest, and established a transcript per million (TPM) of 0 as the cutoff point to include transcripts with low expression levels.

### Gene expression analysis and prognosis

To evaluate gene expression and its association with classical prognostic factors in PCa, we used the OncoDB online database^(24)^ and selected lymph node status, pathological and clinical staging, and survival analysis as clinical evaluation parameters. Expression values were based on RNA-seq. The TCGA population comprised 505 primary tumor samples and 52 adjacent normal tissue samples. Disease-free survival (DFS) analysis was performed using the Gene Expression Profiling Interactive Analysis platform.^(25)^ Kaplan-Meier curves were generated based on the gene expression levels of the PCa samples using the values of the highest and lowest medians as the cutoff points.

### Correlation and functional enrichment analysis

We used the database GEPIA2^(25)^ to investigate the correlations between genes in PCa tumor samples. All expression levels were normalized to those of B2M endogenous controls. To elucidate the biological functions associated with these genes, we conducted pathway enrichment analysis using KEGG (c2 v7.2)^(26)^ and Gene Ontology (GO) (c5 v7.2),^(27,28)^ focusing on biological processes as a parameter. Subsequently, we used the String 12.0 platform^(29)^ to construct the Protein-Protein Interaction networks.

### Statistical analysis

The Mann-Whitney U test was used to evaluate mRNA levels of the prognostic factors. For analyses involving more than two groups, we used ordinary one-way ANOVA and the Kruskal-Wallis test with Bonferroni and Dunn corrections, respectively. We exported data from the OncoDB and GEPIA2 platforms to reanalyze the results and used tests to identify and remove outliers (ROUT [Q=1%]). The Shapiro-Wilk test was used to assess the normality of the data. All graphs were generated, and statistical analyses were performed using GraphPad Prism software (version 9.0). The level of statistical significance was set at 5% (p*<*0.05).

## RESULTS

### miR-137 is underexpressed in tumor tissues and associated with a worse prognosis

**Figure 1:**
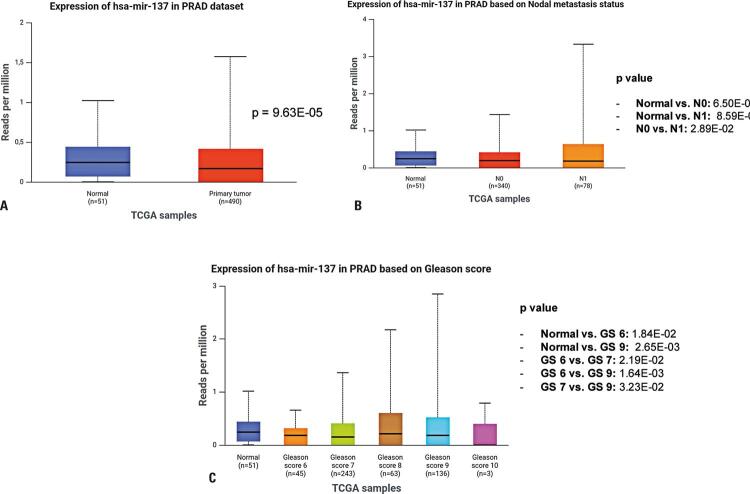
Expression of miR-137 in the prostate cancer TCGA cohort and clinical prognosis. (A) miR-137 levels in prostate cancer and normal tissue. (B) Involvement of regional lymph nodes. (C) Gleason score PRAD: prostate adenocarcinoma; TCGA: The Cancer Genome Atlas; N0: absence of lymph node involvement; N1: Lymph node involvement; GS: Gleason score; NS: non-significant.

Initially, we used UALCAN to examine the miR-137 expression patterns in the PCa samples. We observed a significant underexpression of this miRNA in prostate tumor tissues compared to their respective controls (p*=*9.6E-05; Figure 1A). When analyzing miR-137 expression in the presence or absence of a metastatic nodule, patients with nodular disease had lower expression (p*=*2.90E-02; Figure 1B). Concerning the Gleason score (GS), there were statistically significant differences in miR-137 expression between the Normal, GS6, GS7, and GS9 groups (Figure 1 and Table 1). We observed reduced miR-137 expression in the GS6 and GS9 groups (p<0.05) (Figure 1C). miR-137 expression is associated with cell dedifferentiation in this context. In tumor tissue, there was a significant decrease in miR-137 expression in the GS7 group than in the GS6 group (p<0.05, Figure 1C). Although GS9 had median values like those of GS6, its expression was significantly higher for miR-137 (Figure 1C), which was also observed when compared to GS7 (p<0.05).

**Table 1:** Descriptive statistics related to miR-137 expression levels in association with clinicopathological parameters

Sample types	Median	Min.	Max.
	(Q1-Q3)		
Normal	0.247 (0.072 – 0.434)	0	1.020
Primary tumor	0.170 (0 – 0.413)	0	1.576
Nodal metastasis status			
Normal	0.247 (0.072 – 0.434)	0	1.02
N0	0.191 (0 – 0.409)	0	1.43
N1	0.184 (0 – 0.625)	0	3.325
Gleason score			
Normal	0.247 (0.072 – 0.434)	0	1.020
Gleason score 6	0.182 (0 – 0.308)	0	0.654
Gleason score 7	0.148 (0 – 0.398)	0	1.366
Gleason score 8	0.216 (0 – 0.596)	0	2.175
Gleason score 9	0.185 (0 – 0.514)	0	2.851

Q1: first quartile; Q3: Third quartile; N0: absence of lymph node involvement; N1: lymph node involvement.

### Predictive analysis and behavior of genes in prostate and metabolic tissues

**Figure 2:**
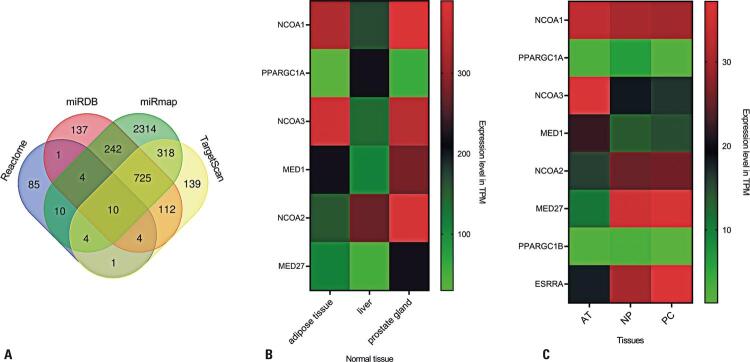
Overview of gene expression in adipose, liver, and prostatic tissues. (A) Reactome, miRDB, miRmap, and TargetScan databases were selected for target prediction analysis of miR-137. The Venn diagram graphically represents the intersection among the platforms, highlighting the overlap of the 10 target genes of the *miRNA*: *PPARGC1A*, *PPARGC1B*, *NCOA1*, *NCOA2*, *NCOA3*, *MED1*, *MED27*, *CHD9*, *TBL1XR*, and *ESRRA*. (B) Heatmap showing gene expression in the liver, prostate, and adipose tissue. (C) Heatmap evidencing gene expression in adipose, benign, and tumor tissue TPM: transcripts per million; AT: adipose tissue; NP: normal prostate; PC: prostate cancer; NS: non-significant.

Subsequently, we analyzed the PPARα gene pathway, which regulates lipid metabolism, using the Reactome database. A total of 119 active genes were identified in this pathway. We performed *in silico* prediction analysis using four databases: Reactome, miRDB, miRmap, and TargetScan. This investigation predicted that 10 genes were miR-137 targets at the intersections between platforms (Figure 2A). The genes included were *PPARGC1A*, *PPARGC1B*, *NCOA1*, *NCOA2*, *NCOA3*, *MED1*, *MED27*, *CHD9*, *TBL1XR*, and *ESRRA*. We verified results using only genes with documented miR-137 interactions (*in vitro* and *in vivo*). Therefore, we focused on the following eight genes: *PPARGC1A*, *PPARGC1B*, *NCOA1*, *NCOA2*, *NCOA3*, *MED1*, *MED27*, and *ESRRA*.

We evaluated the expression of these genes in three tissues using the Expression Atlas database (Table 1S, Supplementary Material). In addition to the prostate, we analyzed the liver and adipose tissues, both of which are characterized by intense lipid metabolism. Heatmap analysis revealed similar expression patterns between the genes in the examined tissues, especially between adipose and prostatic tissues (Figure 2B). Thus, when comparing data from the OncoDB database, we observed that the median expression levels of the genes in benign prostate samples and tumors were similar (Figure 2C).

### Identification of gene expression profile and its association with clinical phenotypes

**Figure 3:**
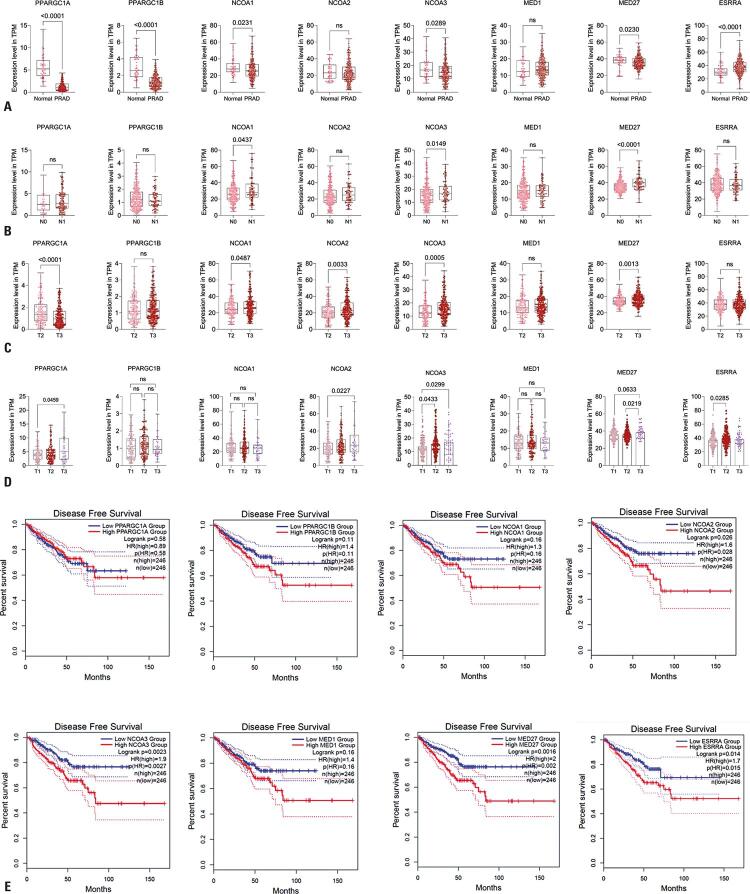
Prognostic value of gene expression in PCa. (A) Gene expression between PRAD and normal tissues. (B) Involvement of lymph nodes. (C) Pathological staging. (D) Clinical staging of the tumor. (E) Kaplan–Meier curve of disease-free survival Statistical significance was set at p<0.05. PRAD: prostate adenocarcinoma; TPM: transcripts per million; NS: non-significant.

Concerning the characteristics of PCa; *PPARGC1A*, *PPARGC1B*, *NCOA1*, *NCOA3*, and MED27 showed attenuated expression profiles compared to normal tissues, with reductions of 0.2-fold, 0.4-fold, 0.8-fold, 0.8-fold, and 0.9-fold, respectively. In contrast, *ESRRA* expression was higher in tumor tissues than in normal tissues (p<0.0001), with a 1.1-fold increase (p*<*0.05, Figure 3A).

When analyzing the expression of these genes in patients with and without regional lymph node involvement, we observed an increase in the expression of *NCOA1*, *NCOA3*, and MED27 in patients with nodular invasion (p*<* 0.05; Figure 3B).

Regarding pathological staging, we observed a decrease in *PPARGC1A* expression and an increase in *NCOA1*, *NCOA2*, *NCOA3*, and MED27 expression in patients classified as T3 compared to those classified as T2 (p*<*0.05; Figure 3C).

Regarding clinical staging, we demonstrated a pattern of positive expression of *NCOA2* and *ESRRA* in patients classified as T2 compared to T1, as well as increased expression of *PPARGC1A*, *NCOA2*, *NCOA3*, and MED27 in those classified as T3 compared to T1. Finally, we identified positive regulation of *ESRRA* in patients with clinical stage T2 compared to those with stage T1 (p*<*0.05, Figure 3D).

In addition, when investigating the relationship between gene expression and DFS, we found an inverse relationship between the time to recurrence and the expression of *NCOA2* (p=0.026), *NCOA3* (p=0.002), MED27 (p=0.001), and *ESRRA* (p=0.014) (Figure 3E).

### Biological functions and interaction profile

**Figure 4:**
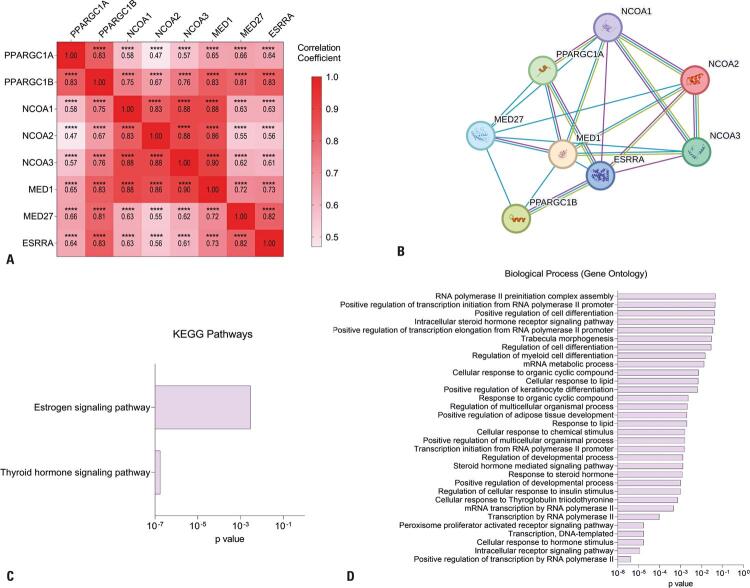
Protein-protein interaction network and enrichment of KEGG pathways of miR-137 target genes. (A) Heatmap representing the correlation matrix between genes in the multivariate analysis. The *B2M* gene was used to normalize all gene expression data. Statistical analyses were performed using the non-parametric Spearman test. ****p*<*0.0001. (B) Stained nodes represent proteins, and the edges of the network correspond to their interactions. Blue lines represent interactions documented in the databases. Pink lines represent experimentally validated interactions. Green lines show protein interactions, based on how often studies report them working together. (C) KEGG pathway enrichment analysis demonstrated increased gene involvement in pathways significantly associated with thyroid hormone and estrogen signaling pathways. (D) Analysis of functional enrichment according to Gene Ontology according to participation in biological processes

In the multivariate analysis, our correlation matrix revealed that all eight genes in PCa exhibited significant positive correlations with each other. *PPARGC1A*, *PPARGC1B*, *NCOA1*, *NCOA2*, *NCOA3*, *MED1*, MED27, and *ESRRA* exhibited distinct expression profiles in cancerous and non-cancerous tissues. However, when the analysis was restricted to tumor tissues, it became evident that these genes were significantly correlated with each other (Figure 4A). Genes exhibit correlations in their expression, indicating possible interactions between them. We hypothesized this phenomenon may be associated with tumor progression.

To better understand the correlations between gene expression in PCa, we used the STRING tool, which displays the interactions between them (Figure 4B). Next, we performed Kyoto Encyclopedia of Genes and Genomes (KEGG) pathway enrichment analysis. The enriched and statistically significant pathways identified were the Estrogen Signaling Pathway and thyroid hormone stimulus (Figure 4C). Moreover, these genes were involved in various ontologies, with notable metabolic, lipid, and hormonal enrichment (Figure 4D).

These results emphasize the complexity of the signaling networks and molecular interactions involved in PCa progression. Given diverse pathways and functions, further investigation of these interactions within PCa is crucial. Such research could provide crucial insights into the development of more targeted and effective therapeutic approaches for treating this condition.

## DISCUSSION

We proposed to endorse our study by investigating *in silico* and literature data of miR-137 target genes associated with PCa progression and lipid metabolism via PPARα. Although most cancers primarily use the glycolytic pathway, evidence suggests that PCa is more likely to use lipid metabolism for survival.^(16)^ Understanding the molecular nuances between early tumors and their progression is crucial for developing more effective and personalized therapeutic strategies.^(30)^

Our findings indicate that miR-137 expression does not follow a linear decrease with tumor progression, but exhibits a more complex pattern. We observed higher levels in normal prostate tissue, followed by a reduction in Gleason scores of 6 and 7. Gleason 9 showed an increase compared to Gleason 6 and 7, although the levels remained lower than those in normal tissues. This variation suggests that, as the tumor becomes less differentiated, miR-137 expression is dysregulated, possibly reflecting an adaptive mechanism or specific characteristic of aggressive tumors.

Because the methylation status of miR-137 in PCa is well established, its epigenetic loss has been associated with post-prostatectomy recurrence and a subset of metastatic samples.^(31)^ This suggests a specific context for the loss of its suppressive function in androgen signaling by modulating the expression of the AR transcriptional co-regulator network. Our group recently demonstrated that restoring this miRNA in a CRPC *in vivo* model and in hypercholesterolemic mice engrafted with PC-3 cells repressed serum and intratumoral cholesterol levels while regulating tumor growth.^(15)^ Thus, epigenetic loss of this molecule is associated with multiple processes involving metabolism, proliferation, invasion, and therapeutic resistance,^(32-35)^ corroborating its suppressive and metabolic roles.

Although PCa is highly heterogeneous, some features become predominant as the disease progresses to an incurable stage. Cancer cells become progressively dependent on the energy content of lipids to ensure their survival,^(36,37)^ thus justifying our findings of elevated gene expression at more advanced stages of cancer.

Considering the scarcity of literature on some of the evaluated genes, we attempted to establish a relationship with tissues that, in addition to being metabolizers, could influence PCa. Adipose tissue directly interacts with cancer cells through adipocytes, enhancing androgenic action. Androgens stimulate lipogenesis *de novo*, promote lipid droplet accumulation and increase fatty acid uptake.^(38-40)^

Although most of the analyzed genes were underexpressed in the primary tumor, they were significantly associated with advanced stages of malignancy, and *PPARGC1A* expression did not differ. According to our results, *PPARGC1A* was underexpressed in tumor tissue samples. However, this increase was only observed in non-organ-confined diseases (pT3). Such an association with advanced disease is clinically concerning as it corroborates the intrinsic relationship between advanced stages and lipid-dependent energy.^(41)^

Xing et al. demonstrated that the miR-137-mediated reduction in *PPARGC1A* levels influences an increase in fat deposits.^(42)^ This regulation is significant and can affect several cellular levels. By inferring this finding for PCa survival, we can hypothesize that its contribution to increased lipid deposition involves the accumulation of fatty acids that, when released from these deposits, bind to and activate *PPARGC1A* in the mitochondria, where they are oxidized to produce sufficient energy to sustain tumor proliferation. Like fatty acids, cholesterol can also dissociate from these deposits and indirectly activate androgen signaling and AR, where *PPARGC1A* functions as a coactivator.^(41)^

In PCa, an increase in *ESRRA* activity is linked to the presence of bone metastases and lower overall survival among patients who relapse after hormone therapy.^(43)^ Our analysis showed higher levels in PCa tissues than in normal samples, and this increase was associated with advanced forms of the disease. These findings are consistent with those of other authors who have reflected on the contribution of *ESRRA* to the initiation and progression of PCa.^(44,45)^ However, no study has evaluated the regulatory influence of the *ESRRA*/miR-137 axis in PCa. Zhao et al. highlighted the intense invasive and migratory capacities of *ESRRA*-positive breast cancer cells. In addition, both of these effects were inhibited in response to miR-137.^(32)^ Our group recently observed that the miR-137 displayed a regulatory impact on the same cellular processes where *ESRRA* participates in PC-3 cells,^(15)^ re-strengthening the need to investigate further the association of miR-137 with genes involved in the PPARα pathway.

Patients receiving hormonal therapy have higher survival rates. However, a chain of drug-induced metabolic events, such as hypercholesterolemia, may be associated with a loss of treatment response, as acquired resistance restores androgen signaling. Thus, the presence of cholesterol results in *ESRRA* activation and its ability to associate with its cofactor *PPARGC1B*, rendering it functionally active and essential for disease progression and resistance to castration.^(46,47)^ We demonstrated that *PPARGC1B* was reduced in the primary tumor and postulated that methylation events in its promoter region confer an increased risk of prostate cancer among patients with telomere shortening.^(48)^ Sahin et al. postulated that *PPARGC1B* was immediately repressed during telomeric dysfunction.^(49)^ Lin et al., in their study, discovered that the metastatic strains DU145 and PC-3, associated with advanced forms of the disease, exhibited elevated levels of telomerase.^(50)^ Subsequently, our group demonstrated that TERF1, which inhibits telomerase activity and regulates telomere length, was downregulated in both cell lines.^(51)^ Thus, these findings lead us to question the fundamental importance of *ESRRA* during the initiation phase of the disease. Because the conditions for its activation and action involve a context-dependent scenario, the functional and structural restoration of telomeres usually occurs with disease progression, where elevated cholesterol concentrations can positively influence the restoration and interaction of *ESRRA* and *PPARGC1B* to maintain the metastatic phenotype.

Although further studies are required, miR-137 may negatively regulate the *ESRRA*/*PPARGC1B* axis in PCa. This modulation can occur at the metabolic and androgenic levels and affect cellular processes related to metastasis, revealing its therapeutic potential.

In our analysis of the p160 family, all three coactivators showed reduced expression in the tumor tissue samples. Despite these findings, there is no evidence of their downregulation in primary tumors. However, increased levels of *NCOA2* and *NCOA3* were associated with reduced DFS, which is consistent with the results of previous reports.^(52,53)^ Furthermore, we identified that the expression of both is related to clinical T3 staging, consistent with earlier findings by our group.^(54)^ Therefore, even at low levels in the primary tumor, these genes can predict the clinical evolution of the disease from its initial presentation, mainly due to the accumulation and direction of their functions, including metabolic functions, to promote PCa progression.

Dasgupta et al. observed that *NCOA2* was highly expressed in metastatic tumors.^(55)^ Our group also demonstrated an inverse relation between *NCOA1* levels and time to metastases.^(54)^

Metastasis requires tumor cells to establish themselves in favorable locations for their survival. Prostate cancer cells exhibit tropism in the marrow owing to the composition of bone tissue, which is enriched in adipose tissue and adipocytes. Several studies have shown that tumor cells capture lipids secreted by marrow adipocytes and use them as a nutritional source for proliferation.^(56-59)^Accordingly, Thysell et al. demonstrated that bone metastases have an abundant cholesterol content, which allows for differentiation from normal bone tissue.^(60)^ Our group demonstrated that supplementation of PC-3 cells with cholesterol increased *NCOA1*, *NCOA2*, and *NCOA3* levels, contributing to the nuclear translocation and colocalization of these coactivators with the (AR).^(6)^ Such events are compatible with aggressive and metastatic phenotypes driven by androgen reactivation in a hypercholesterolemic environment.

These findings are consistent with the role of *NCOA3* in lipid storage, which serves as an energy reserve for cell survival.^(61)^ Independently, *NCOA2* uses intratumoral cholesterol as a precursor for androgen biosynthesis.^(62)^ When associated with *NCOA3*, it induces *de novo* lipogenesis, a non-dietary anabolic process. It also influences the adipogenic differentiation of bone marrow and adipose tissue, mediating the transcriptional activity of PPARy.^(63)^ Finally, *NCOA1* is responsible for mediating lipolysis in differentiated adipocytes so that tumor cells can capture the released fatty acids and produce energy for their proliferation via autocrine mechanisms.^(64,65)^ Thus, we postulated these coactivators influence prostate cancer by stimulating adipogenesis, de novo lipogenesis, and cholesterogenesis, just as the lipid environment favors the presence of coactivators that trigger disease progression.

Our hypothesis is not merely a rehabilitation of existing knowledge. We considered miR-137 to be a key mediator in the regulation of metabolic processes that influence prostatic tumorigenesis. Evidence suggests that the epigenetic modulation by miR-137 negatively affects adipogenesis.^(34,42,66)^ and osteogenic differentiation.^(67-69)^Metastatic PCa cells can interact with cells derived from osteogenesis, such as osteoclasts and osteoblasts, to colonize and promote their growth in the bone metastatic microenvironment.^(59)^ Thus, the presence of miR-137 in this context further underscores its potential as a therapeutic modulator owing to its various molecular actions, especially concerning the prospect of new treatments for patients with lethal prognoses, such as metastasis. Through its restoration, our group observed a more substantial reduction in tumor growth after the suppression of AR activity, mediated by the underexpression of the p160 coactivator family.^(12,13)^ To establish a causal relationship between increased intratumoral cholesterol and AR hyperactivation, we must recognize the existence of an extensive network of oncogenic and metabolic pathways that act in an orderly manner. The literature documented in this study highlights the role of the p160 family in catabolic, anabolic, and tumorigenic processes, and its potential modulation by miR-137 to attenuate the proliferation of malignant cells.^(15,34)^

**Figure 5:**
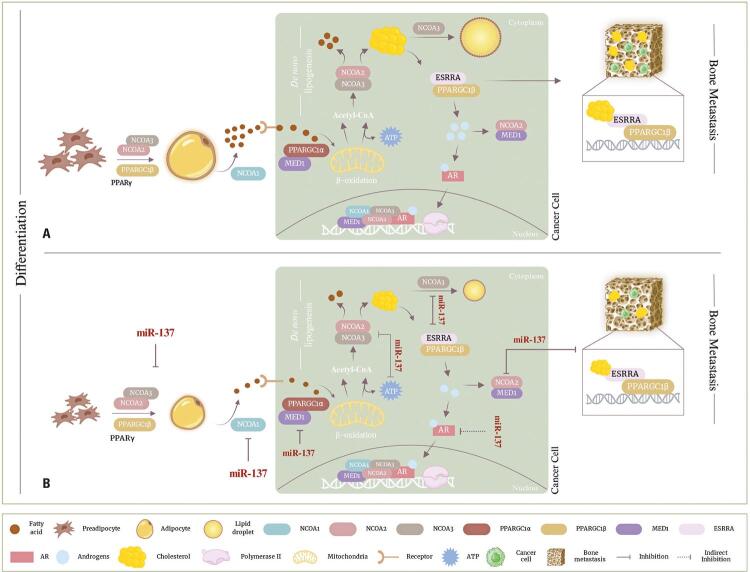
Metabolic reprogramming of prostate cancer cells by miR-137. A) Schematic representation of the maintenance of prostate tumorigenesis sustained by the basal activity of a set of coregulators potentially involved in the oncogenic triad of prostate cancer, cyclically represented by adipogenesis, lipid metabolism, and androgen signaling due to the silencing of miR-137 functions as a transcriptional regulator, resulting from methylation events in its promoter region that induce its epigenetic loss. B) Our hypothesis is that the restoration of miR-137 prevents the occurrence of pro-tumoral events that contribute to the survival of aggressive and metastatic prostate cancer Prepared with the BioRender tool. Available from: https://www.biorender.com/

Our study proposes a mechanism that establishes miR-137 as a central mediator capable of interfering with the crosstalk between metabolic pathways and PCa pathogenesis. Figure 5 illustrates the synthesis involved in this complex, where our results showed that the genes involved in the PPARα lipid pathway have a similar expression profile between metabolizing tissues and tumor tissue and are associated with unfavorable clinical features in PCa. In addition, the correlations between them suggest that the sum of their functions affects tumor progression by mediating adipogenic, lipogenic, and androgenic processes.

## CONCLUSION

Our analysis revealed that miR-137 underexpression was associated with unfavorable prognostic factors, whereas overexpression of its targets was correlated with adverse clinical outcomes, highlighting their crucial roles in prostate cancer progression. We propose a model based on miR-137-mediated regulation, considering its predicted targets in androgen receptor activity and adipocyte metabolism. Theoretically, our study establishes significant metabolic relationships about oncogenic actions in prostate cancer, warranting further investigation and validation to better explore their activity.

## SUPPLEMENTARY MATERIAL


