# Supplementary material for: Interconnected study of molecular pathways: miR-137 as a central element at the intersection of lipid metabolism and prostate carcinogenesis
Source: Einstein (Sao Paulo). 2025 Aug 8;23:eAO1601. doi: 10.31744/einstein_journal/2025AO1601 (PMC12539832; doi:10.31744/einstein_journal/2025AO1601)
Supplement: Supplementary file 1 [file 2317-6385-eins-23-eAO1601-suppl01.pdf]

SUPPLEMENTARY MATERIAL

Interconnected Study of Molecular Pathways: miR-137 as a central element at the intersection of lipid metabolism and prostate carcinogenesis

Karina Serafim da Silva, Vanessa Ribeiro Guimarães, Feres Camargo Maluf, Gabriel Arantes dos Santos, Juliana Alves de Camargo, Iran Amorim da Silva, Katia Ramos Moreira Leite, Sabrina Thalita dos Reis, Nayara Izabel Viana, Miguel Srougi, Ruan Pimenta

DOI: 10.31744/einstein_journal/2025AO1601

Table 1S. Expression atlas

Gene ID	Gene name	Adipose tissue	Liver	Prostate gland
ENSG00000001167	*NFYA*	22.0	14.0	42.0
ENSG00000005339	*CREBBP*	20.0	9.0	26.0
ENSG00000005471	*ABCB4*	0.8	102.0	0.5
ENSG00000008838	*MED24*	18.0	7.0	36.0
ENSG00000012504	*NR1H4*	0.1	84.0	0.3
ENSG00000023330	*ALAS1*	59.0	665.0	64.0
ENSG00000025434	*NR1H3*	52.0	20.0	14.0
ENSG00000042429	*MED17*	1.0	0.2	2.0
ENSG00000054118	*THRAP3*	100.0	63.0	173.0
ENSG00000063322	*MED29*	40.0	23.0	70.0
ENSG00000063438	*AHRR*	0.2		0.6
ENSG00000065833	*ME1*	64.0	13.0	29.0
ENSG00000066136	*NFYC*	17.0	13.0	33.0
ENSG00000069667	*RORA*	9.0	13.0	10.0
ENSG00000072310	*SREBF1*	30.0	15.0	70.0
ENSG00000079459	*FDFT1*	57.0	40.0	103.0
ENSG00000082014	*SMARCD3*	18.0	1.0	31.0
ENSG00000084676	*NCOA1*	33	17	39
ENSG00000099917	*MED15*	18.0	3.0	23.0
ENSG00000100393	*EP300*	25.0	14.0	38.0
ENSG00000101255	*TRIB3*	6.0	16.0	14.0
ENSG00000101849	*TBL1X*	13.0	10.0	48.0
ENSG00000104973	*MED25*	7.0	2.0	12.0
ENSG00000105085	*MED26*	3.0	1.0	5.0
ENSG00000105398	*SULT2A1*		1137.0	
ENSG00000106459	*NRF1*	12.0	4.0	18.0
ENSG00000106546	*AHR*	20.0	43.0	15.0
ENSG00000108510	*MED13*	23.0	26.0	30.0
ENSG00000108590	*MED31*	5.0	3.0	11.0
ENSG00000109819	*PPARGC1A*	3	22	6
ENSG00000110090	*CPT1A*	31.0	86.0	52.0
ENSG00000110243	*APOA5*		143.0	
ENSG00000112237	*CCNC*	25.0	32.0	54.0
ENSG00000112282	*MED23*	17.0	7.0	38.0
ENSG00000112584	*FAM120B*	24.0	9.0	44.0
ENSG00000112972	*HMGCS1*	27.0	142.0	82.0

continue...

...Continuation

Table 1S. Expression atlas

Gene ID	Gene name	Adipose tissue	Liver	Prostate gland
ENSG00000113161	*HMGCR*	13.0	56.0	60.0
ENSG00000115641	*FHL2*	13.0	5.0	48.0
ENSG00000117054	*ACADM*	83.0	198.0	53.0
ENSG00000118137	*APOA1*	0.6	11075.0	1.0
ENSG00000118579	*MED28*	14.0	6.0	25.0
ENSG00000120837	*NFYB*	36.0	17.0	57.0
ENSG00000123066	*MED13L*	15.0	3.0	14.0
ENSG00000123689	*G0S2*	168.0	101.0	35.0
ENSG00000124151	*NCOA3*	37	14	34
ENSG00000124641	*MED20*	13.0	8.0	25.0
ENSG00000125686	*MED1*	22	11	28
ENSG00000126368	*NR1D1*	7.0	12.0	25.0
ENSG00000127511	*SIN3B*	24.0	4.0	44.0
ENSG00000130304	*SLC27A1*	11.0	2.0	20.0
ENSG00000130589	*HELZ2*	8.0	2.0	7.0
ENSG00000130772	*MED18*	11.0	10.0	13.0
ENSG00000131408	*NR1H2*	34.0	12.0	61.0
ENSG00000132170	*PPARG*	155.0	10.0	5.0
ENSG00000132522	*GPS2*	3.0	0.7	4.0
ENSG00000132964	*CDK8*	11.0	10.0	17.0
ENSG00000133398	*MED10*	43.0	12.0	49.0
ENSG00000133794	*ARNTL*	5.0	2.0	5.0
ENSG00000133997	*MED6*	10.0	5.0	15.0
ENSG00000134240	*HMGCS2*	2.0	1945.0	99.0
ENSG00000134317	*GRHL1*	1.0	4.0	15.0
ENSG00000134852	*CLOCK*	12.0	7.0	19.0
ENSG00000135218	*CD36*	1272.0	19.0	4.0
ENSG00000135744	*AGT*	13.0	808.0	18.0
ENSG00000136146	*MED4*	45.0	26.0	60.0
ENSG00000137574	*TGS1*	23.0	8.0	29.0
ENSG00000139278	*GLIPR1*	8.0	5.0	20.0
ENSG00000140396	*NCOA2*	16	27	38
ENSG00000140465	*CYP1A1*	0.2	58.0	
ENSG00000141026	*MED9*	6.0	5.0	12.0
ENSG00000141027	*NCOR1*	21.0	21.0	35.0
ENSG00000142453	*CARM1*	20.0	6.0	31.0
ENSG00000143344	*RGL1*	45.0	17.0	38.0
ENSG00000143437	*ARNT*	37.0	35.0	39.0
ENSG00000146072	*TNFRSF21*	140.0	13.0	51.0
ENSG00000146426	*TIAM2*	0.9	0.4	5.0
ENSG00000147872	*PLIN2*	153.0	361.0	18.0
ENSG00000148297	*MED22*	9.0	2.0	22.0
ENSG00000148677	*ANKRD1*	1.0	2.0	3.0
ENSG00000149485	*FADS1*	15.0	29.0	23.0
ENSG00000151726	*ACSL1*	944.0	931.0	167.0
ENSG00000152944	*MED21*	20.0	13.0	56.0
ENSG00000155111	*CDK19*	11.0	5.0	67.0
ENSG00000155846	*PPARGC1B*	2	0,9	4

continue...

...Continuation

Table 1S. Expression atlas

Gene ID	Gene name	Adipose tissue	Liver	Prostate gland
ENSG00000155868	*MED7*	14.0	6.0	20.0
ENSG00000156603	*MED19*	12.0	5.0	20.0
ENSG00000157184	*CPT2*	4.0	12.0	7.0
ENSG00000158874	*APOA2*	1.0	8065.0	
ENSG00000159479	*MED8*	30.0	18.0	40.0
ENSG00000160563	*MED27*	11	5	22
ENSG00000161533	*ACOX1*	48.0	165.0	47.0
ENSG00000161920	*MED11*	13.0	9.0	20.0
ENSG00000163586	*FABP1*	1.0	2008.0	0.9
ENSG00000164758	*MED30*	17.0	14.0	26.0
ENSG00000165029	*ABCA1*	47.0	31.0	11.0
ENSG00000166821	*PEX11A*	50.0	39.0	48.0
ENSG00000167772	*ANGPTL4*	78.0	83.0	10.0
ENSG00000167910	*CYP7A1*	0.2	34.0	0.1
ENSG00000169375	*SIN3A*	15.0	9.0	27.0
ENSG00000170485	*NPAS2*	1.0	0.5	7.0
ENSG00000171720	*HDAC3*	22.0	16.0	39.0
ENSG00000172379	*ARNT2*	1.0	0.3	15.0
ENSG00000173153	*ESRRA*	18.0	11.0	24.0
ENSG00000175221	*MED16*	16.0	7.0	35.0
ENSG00000177200	*CHD9*	16.0	9.0	25.0
ENSG00000177565	*TBL1XR1*	26.0	22.0	56.0
ENSG00000180182	*MED14*	12.0	13.0	26.0
ENSG00000184634	*MED12*	23.0	5.0	35.0
ENSG00000185591	*SP1*	47.0	19.0	63.0
ENSG00000186350	*RXRA*	51.0	21.0	31.0
ENSG00000186951	*PPARA*	14.0	24.0	15.0
ENSG00000187048	*CYP4A11*	0.2	543.0	
ENSG00000188786	*MTF1*	11.0	3.0	12.0
ENSG00000196498	*NCOR2*	28.0	6.0	46.0
ENSG00000198431	*TXNRD1*	33.0	35.0	68.0
ENSG00000198646	*NCOA6*	15.0	7.0	23.0
ENSG00000198911	*SREBF2*	30.0	24.0	82.0
ENSG00000204231	*RXRB*	37.0	16.0	60.0
ENSG00000241119	*UGT1A9*		45.0	

Query: Genes matching: 'R-HSA-400206', specifically expressed above the expression level cutoff: 0 TPM in experiment E-MTAB-2836.
Selected columns: 32 (all).
Timestamp: Mon, 27-Nov-2023 15:13:26.
